# Multidisciplinary Management of Cerebellopontine Angle Tumors with Brainstem Involvement

**DOI:** 10.3390/audiolres15060168

**Published:** 2025-12-04

**Authors:** Concheri Stefano, Vito Pontillo, Alberto D’Amico, Stefano Di Girolamo, Francesco Signorelli, Elisabetta Zanoletti, Nicola Antonio Adolfo Quaranta

**Affiliations:** 1 Section of Otorhinolaryngology, Department of Neuroscience, University of Padova, Via Giustiniani 2, 35121 Padova, Italy; 2 Otolaryngology Unit, Department of Translational Biomedicine and Neurosciences (DiBraiN), University ‘Aldo Moro’ of Bari, 70124 Bari, Italy; 3 Academic Neurosurgery, Department of Neuroscience, University of Padova, 35121 Padova, Italy; alberto.damico@unipd.it; 4 Department of Otolaryngology, University of Rome “Tor Vergata”, 00133 Rome, Italy; 5 Academic Neurosurgery, University ‘Aldo Moro’ of Bari, 70124 Bari, Italy

**Keywords:** vestibular schwannoma, meningioma, paraganglioma, brainstem, cerebellopontine angle, multidisciplinary management

## Abstract

**Background/Objectives:** Tumors of the cerebellopontine angle (CPA) encompass a limited range of histologies, predominantly vestibular schwannomas (VSs), meningiomas, and paragangliomas (PGLs). Their growth region threatens the cranial nerves (V–XII), brainstem, and cerebellum, possibly causing functional deficits. This review aims to synthesize clinical features and multidisciplinary treatment strategies for CPA tumors with brainstem involvement, emphasizing functional preservation alongside tumor control. **Methods**: A systematic PubMed search identified studies on VSs, CPA meningiomas, and intradural PGLs. Eligibility criteria included studies reporting tumor management and cranial nerve outcomes. Data extraction focused on tumor size, neurological presentation, surgical approach, adjunctive therapies, and postoperative cranial nerve function. Multidisciplinary involvement and rehabilitation strategies were noted. **Results**: Twenty studies (3311 patients) analyzed large VSs, showing facial nerve dysfunction in 8–53%, trigeminal neuropathy in 20–77%, and cerebellar signs in up to 79%. Microsurgery (MS) achieved variable gross total resection, while stereotactic radiosurgery (SRS) preserved facial nerve function but carried trigeminal and hydrocephalus risks. CPA meningiomas demonstrated cranial nerve displacement patterns critical for surgical planning, with transient deficits common and recovery linked to baseline function. In 388 intradural PGL cases, staged surgery combined with preoperative embolization was standard; functional preservation of lower cranial nerves was often limited. Across all histologies, multidisciplinary management and targeted rehabilitation were essential. **Conclusions**: Optimal CPA tumor management balances tumor control with functional preservation. VSs benefit from individualized MS or SRS based on size and mass effect. Meningioma surgery prioritizes cranial nerve preservation over radical resection. Intradural PGLs require staged vascular-conscious approaches. Multidisciplinary care and structured rehabilitation are pivotal to improving outcomes and quality of life.

## 1. Introduction

Tumors of the cerebellopontine angle (CPA) have different but not numerous histologies; they grow in a critical area of interface between bone, brainstem, cerebellum, and brain, where vessels and nerves cross from their origin from the brainstem to the skull base. Their growth, with different patterns, involves an early loss of function of V to XII cranial nerves, which are variably damaged according to the histology, site of origin, and tumor size. The size and pattern of growth are critical and make this tumor at risk of brainstem compression, with peripheral and central neurological sequelae.

Eighth cranial nerve schwannoma is the most frequent primary tumor of the cerebellopontine angle (CPA), followed by meningioma. Epidermoids, VII and V cranial nerve schwannomas, and lipomas are less frequent, whereas secondary growth in the CPA may occur in other tumors of the skull base, e.g., jugular foramen tumors (paraganglioma, schwannoma VS, or meningioma).

Clinical presentation relates to the early or late involvement of cranial nerve function and impact on the brainstem and cerebellum. Function is at risk by tumor growth, histology, and size, as well as by the effects of therapy. Knowledge of the natural history of the disease and the effects of surgery in the CPA, both with traditional and skull base approaches, is paramount.

Typically, a schwannoma of the VIII cranial nerve (vestibular schwannoma (VS)) involves early-onset hearing loss and vestibular symptoms, even in small tumor sizes; further impairment of lower cranial nerves occurs generally later due to CPA growth and brainstem compression. CPA meningiomas account for 2–10% of all intracranial meningiomas and represent the second most common neoplasm of the CPA after vestibular schwannomas [1,2,3]. They originate in different subsites of the CPA and locate anterior, posterior, or eccentric to the internal auditory canal. Early or late involvement of function depends on subsites of growth and size. Contrary to VS, and likely due to the origin from meningeal layers, diagnosis typically occurs at a bigger size, and the onset of cranial nerve impairment occurs later.

Other skull base tumors may grow through the skull base into the CPA, as seen in jugular foramen (JF) paraganglioma (PGL), which extends from the skull base and lower cranial nerve origin to the CPA. The natural history of JF PGL is related to lower cranial nerve impairment and, according to the different subsites of extension, facial nerve and hearing loss. A JF PGL growing in the CPA beyond the posterior fossa dura shows less infiltrative behavior than in bone, but may still produce a consistent mass effect, carry a risk of bleeding, and displace vascular structures and cranial nerves.

The present review aims to summarize the clinical features of large VSs, CPA meningiomas, and intradural PGLs, all growing in the CPA with a consistent mass effect, which requires multidisciplinary management. Our focus is centered on functional aspects related to cranial nerve impairment and brainstem compression. Particular attention is given to cranial nerve deficits beyond hearing, including the facial nerve, trigeminal nerve, and lower cranial nerves, as well as cerebellar and brainstem signs. This review was structured in separate chapters for each histology: VS, meningioma, and intradural PGL.

## 2. Material and Methods

A search of the PubMed database was conducted on August 2025 by a multidisciplinary board of otorhinolaryngologists (ENT), otoneurologists, and neurosurgeons to identify studies reporting large intradural VS, meningioma, and PGL. Duplicates were removed. The remaining studies were carefully screened (through title and abstract examination) to identify those reporting the management of intradural VS, meningioma, or PGL. The screened studies were then assessed for eligibility (through full-text examination) based on the inclusion criteria set for each different histology. The inclusion of case reports and the minimum number of cases in case series were evaluated separately for each histology, based on the number of cases reported in the literature. Technical notes, cadaveric studies, guidelines, radiological classification papers, or non-English papers were excluded. The details of each histology searching methodology are herein reported.

### 2.1. Vestibular Schwannoma

The following string was used for PubMed search: ((“Vestibular Schwannoma”[Mesh] OR “vestibular schwannoma” OR “acoustic neuroma”) AND (“Koos 3” OR “Koos III” OR “Koos 4” OR “Koos IV” OR “large” OR “giant”) AND (“Neurologic Manifestations”[Mesh] OR “neurological symptoms” OR “neurological signs” OR “clinical presentation” OR “sequelae” OR “postoperative complications”[Mesh] OR “postoperative” OR “post-treatment” OR “facial nerve” OR “7th cranial nerve” OR “trigeminal” OR “5th cranial nerve” OR “abducens nerve” OR “6th cranial nerve” OR “lower cranial nerves” OR “glossopharyngeal nerve” OR “9th cranial nerve” OR “vagus nerve” OR “10th cranial nerve” OR “accessory nerve” OR “11th cranial nerve” OR “hypoglossal nerve” OR “12th cranial nerve” OR “cerebellar” OR “ataxia” OR “dysmetria” OR “aphasia”)) AND english[lang] AND (“last 20 years”[PDat]).

Original clinical studies investigating sporadic large or giant VSs, defined according to Koos (grades III–IV) [4] or Tokyo (grades 4–5) [5] classification systems, and reporting neurological symptoms either at presentation or as post-treatment sequelae were included. Eligible studies were required to include at least ten patients and to describe outcomes following microsurgical resection, stereotactic radiosurgery, or combined approaches. Case reports, studies exclusively focused on hearing outcomes and/or facial nerve function, series of small VSs, NF2-related tumors, revision surgeries, or salvage radiosurgery were generally excluded. However, some larger retrospective series that included a minor proportion of patients with Koos I–II tumors, neurofibromatosis type 2 (NF2), or revision surgery/radiosurgery were retained only when neurological symptoms and outcomes were reported separately for the relevant subgroups. This selective inclusion aimed to preserve population homogeneity while avoiding the exclusion of valuable large-sample studies providing relevant clinical information.

### 2.2. Meningioma

The search was performed using combinations of the terms cerebellopontine angle meningioma, petroclival meningioma, cranial nerves, functional outcome, hearing preservation, and diffusion tractography. Emphasis was given to large surgical series, systematic reviews, and consensus guidelines from major societies [6,7,8]. A case report was included because it offered unique anatomical or functional insights [9].

Recent contributions were prioritized to reflect evolving practice: a 2025 Vietnamese series of 36 retrosigmoid resections [10]; a 2024 cohort showing 72.1% symptom improvement but 43.2% transient facial palsy [11]; a 2024 multi-institutional analysis reporting improved gross total resection rates with internal auditory canal drilling [12]; and a 2024/25 endoscopic series achieving a 91.7% rate of long-term facial nerve preservation without new deficits [13].

Additional evidence has recently been provided by Wagner et al., who reported stable or improved cranial nerve outcomes in over 70% of 64 petroclival meningiomas following modern skull base techniques [14]. Similarly, Khaleghi et al. conducted a large meta-analysis of 2884 cases, demonstrating comparable long-term facial nerve results between petroclival and posterior petrous sites, with House–Brackmann grade I–II achieved in over 85% of patients [15]. These findings strengthen the concept of functional preservation as the main determinant of surgical success.

Overall, 25 peer-reviewed studies were synthesized, focusing on the following: (1) anatomical patterns of nerve displacement and vulnerability; (2) implications for surgical approach; (3) prognostic relevance of baseline cranial nerve function; and (4) strategies and adjunctive techniques for nerve preservation [2,16].

### 2.3. Paraganglioma

The search was made combining the string «((“*Paraganglioma*, *Extra-Adrenal*”[*Mesh*]) *NOT* “*Carotid Body Tumor*”[*Mesh*])» with “intracranial” or “intradural” or “class D” or “De” or “Di”. Due to the rarity of intradural PGL, some case reports were included. The following inclusion criteria were set: (i) papers reporting PGLs with intradural extension (i.e., class Di according to Fish) and (ii) reporting data on management. To focus on contemporary management, studies published before 2000 were excluded. Data on tumor management were extracted from the included studies, with particular attention to multidisciplinary approaches and the different specialists considered for tumor treatment, namely geneticists, nuclear medicine physicians, neuroradiologists, surgeons (ENT, otoneurologists, or neurosurgeons), and radiotherapists. The management option chosen for the intradural part of the PGL was separately detailed. The total number of tumors and the number of intradural PGL cases were also recorded. Finally, from each study, it was verified whether the pre- and post-treatment status of the 7–8th and lower cranial nerves was reported.

## 3. Results

### 3.1. Vestibular Schwannoma

#### 3.1.1. Included Studies and Multidisciplinarity

The initial search identified 528 records. After the removal of 12 duplicates and 441 exclusions at the title/abstract screening, 20 studies published between 2010 and 2025 were included in the qualitative analysis, encompassing 3311 patients (Table 1) [17,18,19,20,21,22,23,24,25,26,27,28,29,30,31,32,33,34,35,36]. Of these, 3167 (96%) met the dimensional criteria (Koos III–IV, Tokyo 4–5). Ten studies used the anatomical/topographical Koos classification, seven the dimensional Tokyo classification (or variants), and three applied both. Most series were single-center (90%), with two multicenter collaborations. NF2 cases were mostly excluded, except for small proportions (<10%), as were revision or secondary treatments with no subgroup stratification. A multidisciplinary approach was reported or inferred in 70% of studies, typically involving neurosurgeons, otoneurologists, neuroradiologists, neurologists, and radiation oncologists; neurophysiologists and rehabilitation specialists were only rarely mentioned.

Microsurgery (MS) was the main treatment modality (2385 cases, 16 studies), followed by stereotactic radiosurgery (SRS) (782 cases, 5 studies). Twelve studies reported both pre- and post-treatment neurological symptoms, while eight focused only on either presenting symptoms (n = 4) or treatment sequelae (n = 4).

**Table 1:** Summary of included studies reporting large and giant vestibular schwannomas. All studies are monocentric, except Bretonnier et al. [28] (num. 12) and Pikis et al. [33] (num 17). NS—neurosurgeon; OT—neurotologist; MN—medical neurologist; NR—neuroradiologist; RO—radiation oncologist; NR—neurorehabilitator; MP—medical physicist; NP—neurophysiologist; PS—plastic surgeon; NA—not available; MS—microsurgery; SRS—stereotactic radiosurgery; RSA—retrosigmoid approach; TLA—translabyrinthine approach; LINAC—LINear ACcelerator; GKS—gamma knife surgery; D—dimensional; T—topographical; B—both.

#	Author (s)	Year of Publication	Country	Multidisciplinarity	Specialists	Total Number of Cases	Cases Meeting Dimensional Criteria		Inclusion Criteria	NF2 (%)	Secondary Treatment (%)	Type of Treatment	Neurological Sympoms	
							N	%					Pre	Post
**1**	**Mandl et al. [17]**	2010	The Netherlands	Y	NS, RO	29	29	100%	D	10%	31%	SRS	Yes	Yes
**2**	**Samii et al. [18]**	2010	Germany	N	NS	50	50	100%	D	4%	12%	MS	Yes	Yes
**3**	**Lim et al. [19]**	2013	Malaysia	N	NS	32	27	84%	D	None	NA	MS	Yes	Yes
**4**	**Betka et al. [20]**	2014	Czech Rep.	Y	OT, NS, NR	333	287	86%	T	NA	5%	MS	No	Yes
**5**	**Iorio-Morin et al. [21]**	2016	Canada	Y	NS, RO, MP	68	68	100%	T	None	21%	SRS	Yes	Yes
**6**	**Boublata et al. [22]**	2017	Algeria	N	NS	151	151	100%	D	NA	NA	MS	Yes	Yes
**7**	**Huang et al. [23]**	2017	China	Y	NS, NP, NR, PS	657	657	100%	D	None	NA	MS	Yes	Yes
**8**	**Zumofen et al. [24]**	2017	Switzerland	Y	NS, NR, NP, OT	44	44	100%	T	None	5%	MS	Yes	Yes
**9**	**Troude et al. [25]**	2019	France	Y	NS, OT, RO	169	169	100%	T	6%	18%	MS	Yes	No
**10**	**Anselmo et al. [26]**	2020	Italy	Y	NS, RO, NR	48	21	44%	T	None	43%	SRS	No	Yes
**11**	**Balasa et al. [27]**	2020	Romania	Y	NS, MN	66	66	100%	T	None	NA	MS	Yes	Yes
**12**	**Bretonnier et al. [28]**	2020	France	Y	NS, MN	60	60	100%	D	NA	None	MS	No	Yes
**13**	**Elsayed et al. [29]**	2021	France	Y	NS, NP, OT	43	41	95%	T	None	None	MS	Yes	No
**14**	**Kiyofuji et al. [30]**	2021	USA	Y	OT, NS	86	48	56%	B	8%	6%	MS	Yes	No
**15**	**Mastronardi et al. [31]**	2021	Italy	N	NS	60	60	100%	B	3%	NA	MS	Yes	Yes
**16**	**Wang et al. [32]**	2022	China	N	NS	100	82	82%	B	None	None	MS	Yes	No
**17**	**Pikis et al. [33]**	2023	International	Y	RO, NS	627	627	100%	T	None	None	SRS	Yes	Yes
**18**	**Kamogawa et al. [34]**	2024	Japan	Y	RO, NS	58	58	100%	T	None	None	MS (36%), SRS (64%)	Yes	Yes
**19**	**Almashhadani et al. [35]**	2025	Italy	N	OT	567	567	100%	D	NA	NA	MS	Yes	Yes
**20**	**Tang et al. [36]**	2025	China	Y	NS, MN	63	55	87%	T	None	None	MS	No	Yes
	**Tot.**			**70%**		**3311**	**3167**	**96%**				**2385 MS-782 SRS**	**80%**	**80%**

#### 3.1.2. Demographics and Pathological Aspects (Table 2)

Mean age at diagnosis ranged from late forties to early fifties, with a slight female predominance (overall M/F ratio 0.8).

Tumor classification included 544 Tokyo 4 (“large”), 1105 Tokyo 5 (“giant”), 194 Koos III, and 1350 Koos IV. Tumor size was heterogeneously reported (mean diameter 2.7–4.4 cm; mean volume 7.4–16.5 cm^3^). Cystic morphology was described in 7–50% of cases, particularly in larger cohorts.

#### 3.1.3. Neurological Symptoms at Presentation (Table 2)

Facial nerve dysfunction occurred in 18% of patients overall (range 5–53%). Trigeminal neuropathy was frequent (20–77%); Tang et al. [36] reported a 100% prevalence, although this reflects a selection bias, as only patients with trigeminal symptoms were included in their study. Lower cranial nerve palsy, long tract signs, and oculomotor deficits were less common but more typical of Tokyo 5 and Koos IV tumors. Cerebellar symptoms (imbalance, ataxia, aphasia) and hydrocephalus occurred in up to 79 and 60% of cases, respectively, again mainly in giant tumors with brainstem compression.

#### 3.1.4. Treatment Strategies and Outcomes (Table 2)

Twenty-one studies reported on microsurgery (MS), mostly employing retrosigmoid (1778 cases) and translabyrinthine (560 cases) approaches. Gross total resection (GTR) rates widely varied, with more recent series showing a trend toward conservative resections (50–90% GTR) to preserve neurological function. Five studies addressed stereotactic radiosurgery (SRS), used either as first-line therapy (766 cases) or secondary after incomplete resections (43 cases).

Recurrence/regrowth rates ranged from 1 to 22% after MS and 0 to 13% after SRS. Variability was linked to the extent of resection: cohorts with more subtotal or partial resections showed higher regrowth rates compared with those with mostly near total resections.

#### 3.1.5. Post-Treatment Neurological Sequelae (Table 2)

Poor facial nerve outcome (≥HB III) was the most frequent postoperative complication (average 27%, range 5–58%), especially after GTR. SRS yielded lower rates of permanent facial palsy (0–4%), except in series with larger tumors [17]. Trigeminal dysfunction occurred in 7–22% of MS cases and 16–32% of SRS cases. Lower cranial nerve palsy, oculomotor palsy, long tract signs, and cerebellar/brainstem complications were rare (<8%). Post-treatment hydrocephalus occurred in up to 12% after MS and up to 21% after SRS, usually in giant tumors. Treatment-related mortality was exceedingly low or absent.

### 3.2. Meningioma

#### 3.2.1. Anatomy and Nerve Displacement (Table 3)

The CPA is a compact cistern containing CNs V–XII in close relation to major vascular structures. Even small meningiomas may cause deficits [1,16]. CN V is typically displaced superiorly, explaining trigeminal neuralgia. CN VI, due to its long cisternal course, is highly vulnerable in petroclival tumors [37]. The CN VII–VIII complex is usually displaced anteriorly or inferiorly, though petroclival lesions may push it posteriorly, complicating retrosigmoid access [2]. Lower cranial nerves are rarely affected except in giant lesions; CN XII is compromised mainly in inferior extension [38]. Major arteries (AICA, PICA, basilar perforators) and venous sinuses are frequently displaced or encased [37]. High-resolution MRI, particularly CISS/FIESTA sequences, predicts displacement and supports surgical planning [39].

Recent series have provided more refined data on these anatomical relationships. Do et al. [10] and Mehta et al. [12] correlated specific displacement patterns with postoperative nerve outcomes, emphasizing that nerves compressed rather than infiltrated by tumor have higher recovery potential. Kong et al. further demonstrated that endoscopic visualization can clarify nerve trajectories around the petroclival junction, reducing the risk of inadvertent traction or transection [40].

#### 3.2.2. Surgical Implications

Patterns of nerve displacement dictate dissection strategy. Recognition of CN V or CN VII–VIII thinning reduces traction-related injury [1]. Preservation of vascular loops and venous drainage is crucial. Retrosigmoid craniotomy remains effective for most CPA meningiomas, whereas petroclival tumors—displacing nerves posteriorly—may require combined petrosal or extended presigmoid approaches [41,42,43]. In patients with non-serviceable hearing, the translabyrinthine or transcochlear route is considered [44]. Endoscopic assistance extends visualization into blind corridors [13,45].

Recent data confirm that approach selection directly influences cranial nerve out-comes. Khaleghi et al. demonstrated that petroclival and posterior petrous meningiomas have similar functional results when modern microsurgical principles are applied [15]. Kong et al. [40] and Xie et al. [13] highlighted how endoscopic or combined transorbital routes improve the illumination of deep corridors while maintaining facial and trigeminal nerve integrity. These findings suggest that minimally invasive and hybrid approaches can achieve effective tumor control with reduced neurological morbidity, although they may pose challenges in managing intraoperative issues such as bleeding.

#### 3.2.3. Preoperative Assessment

MRI with gadolinium, FLAIR, and CISS/FIESTA defines tumor margins and displacement; MRA/MRV delineates vascular encasement [8]. CT complements MRI by identifying osseous involvement [46]. Baseline function is documented with standardized tools: Gardner–Robertson/AAO-HNS for hearing, House–Brackmann for facial nerve, and targeted electrophysiological tests for other CNs [47].

Emerging imaging modalities such as diffusion tensor tractography now allow preoperative mapping of cranial nerve trajectories, improving risk prediction and surgical planning [48,49]. Functional MRI and advanced neuronavigation may further delineate tumor–nerve relationships, aiding intraoperative orientation and limiting unnecessary traction.

#### 3.2.4. Prognosis and Outcomes

Baseline function is the strongest predictor of recovery: recent, mild deficits are reversible, while chronic dysfunction rarely improves [50]. Nerves displaced rather than encased fare better.

○ CN V: Surgery often relieves neuralgia, especially if recent [51].
○ CN VII: Dysfunction occurs in 5–10% of CPA and up to 15% of petroclival tumors [1,2]. Transient palsy is common but usually resolves [11].
○ CN VIII: Hearing loss occurs in 20–40% of CPA and up to 60% of petroclival meningiomas; preservation reaches 50–70% when nerves are displaced but not encased [12].
○ CN VI: Palsy is frequent in petroclival lesions (15–20% preoperatively, 25% postoperatively), though often recovers [37].
○ CNs IX–XI: Deficits in up to 20% of giant tumors [7,37].
○ CN XII: Involved in 10–15% of inferior extensions, with limited recovery [38].

Supplementary evidence from Wagner et al. [14] and Pham et al. [52] showed that persistent postoperative cranial nerve deficits, mainly affecting CN VII and VIII, occur in approximately 10–15% of cases, with improved results in centers employing intraoperative monitoring and early rehabilitation. Papazian et al. further confirmed that internal auditory canal invasion predicts poorer auditory outcomes [49].

### 3.3. Paraganglioma

The PubMed search yielded a total of 465 records. After the removal of 29 duplicates, 439 unique studies remained for screening. Title and abstract screening excluded 387 studies that did not meet the inclusion criteria, leaving 52 articles for full-text assessment. Following the full-text review, 14 studies fulfilled all eligibility criteria and were included in the analysis. The included articles consisted of 1 case report and 13 case series (see Table 4), published between 2000 and 2018 [53,54,55,56,57,58,59,60,61,62,63,64,65,66].

Across the included studies, a total of 1506 PGL cases were reported, of which 388 involved intradural extension. It should be noted that some studies may have reported cases that were also included in other studies within the review, potentially leading to a partial overlap of patient data. Data on management were available in all included studies, describing a multidisciplinary management (at least two different specialists) in 13/14 (93%) cases. The most frequently involved specialists were surgeons (14/14), followed by neuroradiologists for angiography and/or angioembolization (12/14) and radiotherapists (10/14). Nuclear medicine physicians for PET execution or geneticists for genetic tests were involved, respectively, in 0/14 and 1/14 of the studies included. Surgery was the management option chosen for the intradural part of the PGL in all the studies.

Regarding cranial nerve status, all studies reported information on pre-treatment function, and 13/14 reported also post-treatment function, specifically for the 7–8th and lower cranial nerves. Data from each study are reported in Table 4.

**Table 2:** Vestibular schwannoma: demographics, pathological aspects, presentation of neurological symptoms, surgical aspects, outcome, and post-treatment neurological sequelae. FP—facial palsy; TN—trigeminal neuropathy; LCN—lower cranial nerve palsy; OCM—oculomotor cranial nerve (3rd-4th-6th) palsy; LTS—long tract signs; CBL—cerebellar signs; HCP—hydrocephalus; MS—microsurgery; RSA—retrosigmoid approach; TLA—translabyrinthine approach; GTR—gross total resection; SRS—stereotactic radiosurgery; LINAC—LINear ACcelerator; GKS—gamma knife surgery.

#	Author, Year	Mean Age	Male/ Female Ratio	Mean Diameter (cm)	Mean Volume (cm^3^)	Tokyo 4 (‘Large’)	TTokyo 5 (‘Giant’)	Koos III	Koos IV	Cystic %	Presentation Neurological Symptoms	Surgical Approach/Type of SRS	GTR %	Re-Growth/Recurrence	Post-Treatment Neurological Symptoms	Mortality	Median Follow-Up (Months)
**1**	**Mandl et al. [17]**	54.1	0.17	3.3	15.3	29	0	-	-	-	28% FP; 41% TN; 3% CBL; 7% HCP	LINAC	-	-	29% FP; 23% TN; 8% LCN; 8% OCM; 21% HCP	-	36 (12–120)
**2**	**Samii et al. [18]**	42.1	0.92	4.4	-	0	50	-	-	-	6% FP; 38% TN; 10% LCN; 4% OCM; 14% LTS; 64% CBL; 26% HCP	RSA	100%	-	25% FP; 2% (+4%) LCN	0	34 (5–62)
**3**	**Lim et al. [19]**	50.4	0.28	3.6	-	27	0	-	-	-	15% FP; 25% TN; 6% LCN; 4% OCM; 4% LTS; 41% CBL; 7% HCP	RSA	44%	-	26% FP; 7% TN; 4% OCM; 4% CBL 4%	0	40.1
**4**	**Betka et al. [20]**	48	0.73	-	-	-	-	62	225	-	3% HCP	RSA 97%-TLA 2%	98%	1%	34% FP%; 1% TN; 6% LCN; 0.3% OCM; 0.3% LTS	3%	12–78
**5**	**Iorio-Morin et al. [21]**	58	1.43	-	7.4	-	-	0	68	19%	9% FP; 36% TN; 7% HCP	GKS	-	-	6% (+9%) TN; 4% HCP	1.5%	47 (6–125)
**6**	**Boublata et al. [22]**	48.2	0.44	-	-	92	59	-	-	7%	5% FP; 20% TN; 3% LCN; 48% CBL; 33% HCP	RSA	83%	-	18% FP; 3% LCN; 2% OCM; 4% CBL	0.7%	28 (3–54)
**7**	**Huang et al. [23]**	46.8	0.79	-	-	0	657	-	-	-	31% FP; 69% TN; 2% HCP	RSA	84.6%	-	44% FP; 16% TN; 7.5% LCN; 7% CBL; 1% HCP	0.6%	60 (6–191)
**8**	**Zumofen et al. [24]**	58	1.59	-	10.9	-	-	0	44	19%	25% FP; 45% TN; 2% OCM; 30% CBL; 11% HCP	RSA	0%	16%	11% FP; 15% TN	0	22 (1–72)
**9**	**Troude et al. [25]**	51	0.66	3	16.5	-	-	0	169	21%	10% FP; 46% TN; 4% LTS; 12% CBL	RSA 64%-TLA 36%	11%	17%	16% FP	-	62 (54–71)
**10**	**Anselmo et al. [26]**	-	-	-	-	-	-	15	6	-	-	LINAC	-	0	14% FP; 24% (+28%) TN; 5% CBL; 5% HCP	-	147
**11**	**Balasa et al. [27]**	52.9	0.50	-	-	-	-	0	66	-	53% FP; 38% TN; 18% LCN and OCM; 79% CBL; 53% HCP	RSA	36%	3%	16% FP; 2% LCN; 2% CBL; 12% HCP	2%	≥60
**12**	**Bretonnier et al. [28]**	-	1.07	4.2	-	0	60	-	-	32%	12% FP	TLA 62%-RSA 38%	35%	22%	43% FP; 8% LCN; 2% LTS; 2% HCP	0%	45
**13**	**Elsayed et al. [29]**	51	0.87	-	-	-	-	27	14	40%	44% TN; 2% LCN; 28% CBL	RSA 81%-TLA 18%	51%	-	5% FP	-	-
**14**	**Kiyofuji et al. [30]**	46.3	1.18	4.3	-	0	48	0	48	-	20% FP; 65% TN; 8% LCN; 12% OCM; 44% CBL; 60% HCP	RSA 90%-TPLA 10%	50%	12%	50% FP	-	59 (0–192)
**15**	**Mastronardi et al. [31]**	48.8	0.76	3.97	-	25	35	0	60	23%	18% FP; 33% TN; 30% CBL	RSA	77%	13%	28% FP; 2% LCN; 2% (+4%) OCM; 4% CBL; 2% HCP	0	59.3 (6–113)
**16**	**Wang et al. [32]**	48.8	0.52	-	-	-	-	34	48	30%	4% TN; 5% CBL; 6% HCP	RSA	90%	-	22% FP	-	-
**17**	**Pikis et al. [33]**	54	0.82	-	8.7	-	-	0	627	-	8% FP; 32% TN; 1% OCM; 0.2% LTS; 13% CBL; 2% HCP	GKS	-	6%	4% FP; 32% TN; 7% CBL; 6% HCP	0	38
**18**	**Kamogawa et al. [34]**	57	0.93	-	-	-	-	58	0	24%	3% TN; 7% HCP	RSA 36%-GKS 64%	76% MS	14% MS | 13% SRS	MS: 9.5% FP; 0 TN; 5% LCN; 5% CBL; 6% HCP-SRS: 0 FP; 16% TN; 0 LCN; 0 CBL; 8% HCP	-	57 MS | 82 SRS
**19**	**Almashhadani et al. [35]**	49.2	0.99	3.6	-	371	196	-	-	50%	7% FP; 77% TN; 11% LCN; 6% OCM; 21% HCP	TLA 94%	73%	6%	58% FP; 1% TN; 0.3% LCN; 3% OCM; 2% CBL	0	50 (12–248)
**20**	**Tang et al. [36]**	56	0.62	2.7	-	-	-	32	23	-	-	RSA	78%	-	13% FP; 22% TN	0	34 (20–50)
	**Tot.**					**544**	**1105**	**194**	**1350**								

**Table 3:** Cranial nerve involvement and functional outcomes in cerebellopontine meningiomas and in petroclival meningiomas.

Cranial Nerve	Preoperative Dysfunction–CPA (%)	Preoperative Dysfunction–Petroclival (%)	Postoperative Outcome	Key References
**CN V**	20–30% (neuralgia, hypoesthesia)	40–50%	Pain relief often achieved if recent; sensory loss rarely recovers	Samii, 1997 [16]
**CN VII**	5–10%	Up to 15%	HB grade I–II in most cases; transient palsy common, permanent deficit uncommon	Nakamura, 2005 [1]
**CN VIII**	20–40% (hearing loss, tinnitus)	40–60%	Hearing preservation in 50–70% with serviceable hearing if displaced; poor if encased	Stavrou, 2008 [50]
**CN VI**	<10%	15–20%	Postop palsy up to 25%; many recover spontaneously	Natarajan, 2007 [37]
**CN IX–XI**	5–10%	15–20%	Dysphagia/hoarseness may persist; risk of aspiration in severe cases	Nakamura, 2005 [1]
**CN XII**	Rare (<5%)	10–15%	Persistent tongue weakness/atrophy common if injured	Agarwal, 2018 [38]

**Table 4:** Summary of included studies reporting intradural PGL cases. All papers reported pre-treatment cranial nerve status. * or ^+^ denote cases that may overlap with others marked with the same symbol. 1—option considered/data reported in the paper; 0—option not considered/data not reported in the paper.

First Author	Total of PGL Cases	Total of Intradural PGL Cases	Genetic Testing	PET	Angiography	Embolization	RT	Surgery	Post-Treatment Report	
									7–8th Cranial Nerves	Lower Cranial Nerves
Mazzoni, 2019 [53]	175 *	63 *	0	0	1	1	1	1	1	1
Sivalingam, 2012 [54]	156 ^+^	55 ^+^	0	0	1	1	1	1	1	1
Shin, 2011 [55]	230 ^+^	7 ^+^	1	0	1	1	1	1	1	1
Sanna, 2011 [56]	212 ^+^	45 ^+^	0	0	1	1	1	1	1	1
Borba, 2010 [57]	32	14	0	0	1	1	0	1	1	1
Mazzoni, 2015 [58]	171 *	63 *	0	0	1	0	1	1	1	1
Mazzoni, 2009 [59]	11 *	4 *	0	0	0	0	0	1	1	1
Mattos, 2004 [60]	6	6	0	0	1	1	0	1	1	1
Prasad, 2015 [61]	185 ^+^	71 ^+^	0	0	1	1	1	1	1	1
Michelozzi, 2015 [62]	18	6	0	0	1	1	1	1	1	1
Sanna, 2004 [63]	53 ^+^	24 ^+^	0	0	1	1	1	1	1	1
Jackson, 2004 [64]	228	22	0	0	1	1	1	1	0	0
Oghalai, 2004 [65]	28	7	0	0	0	1	0	1	1	1
Brewis, 2000 [66]	1	1	0	0	1	1	1	1	1	1
	1506	388	1	0	12	12	10	14	13	13
		26%	7%	0%	86%	86%	71%	100%	93%	93%

## 4. Discussion

Preservation of function, tumor control and, when feasible, cure of disease represent the current paradigm in the management of cerebellopontine angle tumors. In vestibular schwannoma, functional preservation is most strongly associated with surgical removal of small tumors, whereas large size tumors with brainstem compression involve a higher risk of neurological sequelae, both at diagnosis and after surgery. In paraganglioma, the intradural component is generally related to a higher stage disease, with extra-intracranial extension. Lower cranial nerve morbidity is common both in the diagnostic and post-surgical setting.

Specific issues for the three histologies are herein debated.

### 4.1. Vestibular Schwannoma

Most of the literature has focused on hearing preservation and facial nerve outcomes, whereas data on neurological presentation symptoms and sequelae after treatment remain scarce and inconsistently reported. Such outcomes are crucial in large and giant VSs, where mass effect on the brainstem is common, leading to broader neurological morbidity. In this context, multidisciplinary management—radiology, neurosurgery, neurotology, radiation oncology, and rehabilitation—becomes essential for both treatment planning and postoperative care.

In our analysis, facial palsy or neuropathy at presentation reached 53% in some cohorts but was substantially lower (8–12%) when only treatment-naïve series were considered. In contrast, trigeminal neuropathy was more frequent, affecting 20–77% of patients. This likely reflects the greater vulnerability of sensory fibers to compression compared with motor fibers [67]. Other manifestations, such as lower cranial nerve palsy (≤10%) and ocular motor involvement (≤6%), were less frequent but clinically relevant. Notably, LCN or 6th cranial nerve dysfunctions may result from mass effect on the nerve itself or from pressure-related changes associated with intracranial hypertension, while 3rd and 4th cranial nerves are typically protected from direct tumor compression, due to their origin above the CPA. Long tract signs, such as hemiparesis, occurred in ≤14% of cases, mainly in giant or Koos IV tumors. Cerebellar symptoms (imbalance, ataxia) were highly prevalent, ranging from 3% to 79%, and consistently correlated with tumor size. Hydrocephalus was observed in 2–33%, predominantly in giant tumors, confirming the role of fourth ventricle distortion in CSF obstruction. It should be acknowledged that a selection bias exists, as SRS is generally not offered to patients presenting with hydrocephalus, disabling ataxia, trigeminal neuropathy, or refractory headache.

Post-treatment, facial nerve dysfunction was the most frequent sequela, affecting 27% of MS patients compared with 9% of SRS patients. Trigeminal neuropathy occurred in up to 22% after MS and up to 32% after SRS. Microsurgery may relieve trigeminal neuralgia through decompression, with >90% improvement reported in one series, whereas SRS rarely improves symptoms and may even worsen neuropathy [68]. Lower cranial nerve and ocular motor palsies rarely exceeded 8% but could be disabling, requiring complex rehabilitation. Transient imbalance and ataxia were frequent after MS [22,69], though most cases improved over time; permanent cerebellar morbidity was uncommon but severe. Hydrocephalus was more frequent after SRS (up to 21%) than after MS (≤12%), highlighting a distinct late risk associated with radiosurgery in larger tumors.

To summarize, SRS provides excellent tumor control and favorable facial nerve preservation but carries its own risks, particularly trigeminal neuropathy and delayed hydrocephalus. Thus, while SRS is a valuable option for small to medium lesions or postoperative remnants, its role in large and giant VSs with mass effect remains limited. The extent of surgical resection is another key determinant of outcomes. Gross total resection (GTR) is associated with higher rates of facial nerve palsy [27,70], but stratified data on other neurological complications by extent of resection are limited; where reported, no consistent or robust differences emerge as clearly as for facial nerve outcomes. Conversely, NTR strategies offer better functional preservation without compromising short- to mid-term tumor control, particularly when followed by adjuvant SRS [24,71]. These findings support a paradigm shift toward function-preserving surgery in patients with good baseline neurological function.

Despite frequent references to multidisciplinary management, few studies systematically addressed the role of rehabilitation. Long-term cohorts show that deficits such as phonatory dysfunction may persist in up to one-quarter of patients [72], underscoring the need for structured rehabilitation programs, including voice, swallowing, and balance therapy. These interventions are critical for improving quality of life and should be integrated into clinical pathways for patients with VS. Taken together, the current evidence suggests that the management of large and giant VSs should be individualized, balancing tumor control with functional preservation. Optimal care requires collaboration among neurosurgeons, neurotologists, radiation oncologists, neuroradiologists, and rehabilitation specialists. Future studies should adopt standardized outcome reporting, including all cranial nerves, cerebellar and brainstem function, and patient-reported measures. Only through such comprehensive assessment can the true burden of neurological morbidity be fully understood.

Several limitations emerge from the literature review on VS. First, most studies were retrospective, single-institution series, with inherent selection bias. Second, reporting of neurological outcomes was inconsistent: facial nerve data were nearly universal, but other cranial nerve and cerebellar outcomes were often missing. Third, radiosurgery studies generally excluded patients with hydrocephalus or symptomatic brainstem compression, limiting applicability to very large tumors. Fourth, some series included small proportions of NF2 cases or previously treated patients, further complicating comparisons. Finally, long-term data on quality of life and rehabilitation remain scarce. Future research should prioritize prospective designs, standardized reporting, and integration of neurological and functional outcomes into core datasets. Classification systems should be consistently applied, whether Koos, Tokyo, or ideally a unified system, to facilitate comparison across studies. Importantly, functional outcomes—voice, swallowing, balance—should be systematically assessed alongside oncological control. Rehabilitation strategies must become a structured component of management, given their proven impact on long-term outcomes.

### 4.2. Meningiomas

#### 4.2.1. Paradigm Shift in Surgical Goals

Cranial nerve preservation has become the central goal in CPA and petroclival meningioma surgery. The early emphasis on radical excision has been replaced by the principle of maximal safe resection due to high morbidity associated with aggressive approaches [6,7,16]. This shift is strongly supported by recent guidelines from the EANO and large cooperative analyses, which underline the concept of functional cure—achieving durable tumor control while maintaining neurological integrity [6,73]. Quality-of-life studies have also shown that the preservation of cranial nerve function is a better predictor of long-term satisfaction than the extent of resection alone [74].

#### 4.2.2. Determinants of Nerve Preservation

High-resolution MRI and angiography are indispensable for defining nerve displacement and vascular encasement [8]. Baseline neurological function, assessed by validated scales, serves as a strong prognostic marker. Patients with preserved preoperative function have the highest likelihood of recovery, underscoring the importance of early referral. Additional determinants include the plane of the tumor–nerve interface, the degree of arachnoid preservation, and the use of continuous intraoperative neurophysiological monitoring [10]. Evidence suggests that nerve displacement without adherence to tumor capsule correlates with functional recovery, while encasement predicts persistent deficits [12].

#### 4.2.3. Microsurgical Principles

Key technical steps include early devascularization, piecemeal tumor debulking, arachnoid plane preservation, and sharp dissection in areas of nerve thinning [1]. Avoidance of fixed retraction, combined with cerebrospinal fluid release and optimal patient positioning, reduces traction-related injury. Intraoperative neurophysiological monitoring further reduces the risk of permanent deficits [47,75]. Endoscopic assistance improves visualization of the internal auditory canal and petroclival recess [13,46]. Recent refinements include the use of angled endoscopes and 3D visualization, which permit safer dissection in deep corridors [41]. Continuous electromyographic feedback, quantitative facial nerve monitoring, and near-infrared fluorescence imaging have also been proposed as adjuncts to improve intraoperative decision making [15].

#### 4.2.4. Approach Selection

The retrosigmoid approach remains versatile for most CPA meningiomas. Petroclival lesions, which typically displace nerves posteriorly, often require combined petrosal or extended presigmoid approaches [37,41,42]. The translabyrinthine and transcochlear routes are reserved for patients lacking serviceable hearing [44]. Recent comparative studies indicate that tailored combined approaches—retrosigmoid combined with posterior petrosal—can achieve higher rates of resection without increasing cranial nerve morbidity [14,15]. Endoscopic and keyhole variations further expand visualization while minimizing retraction-related injury [40].

#### 4.2.5. Functional Outcomes by Nerve

- CN V: Neuralgia typically improves after decompression; long-standing numbness rarely recovers [51].
- CN VII: Most modern series report long-term House–Brackmann grade I–II outcomes; transient postoperative weakness is common but typically reversible [2,11].
- CN VIII: Hearing preservation rates remain 50–70% in selected cases [12,48].
- CN VI: Palsy is frequent but recovery is common [37].
- CN IX–XI: Often compromised in giant tumors, with persistent postoperative deficits [7,37].
- CN XII: Rarely involved, but when present, deficits are functionally disabling [38].

#### 4.2.6. Adjunctive and Staged Strategies

In giant or adherent tumors, subtotal resection followed by radiosurgery provides a safe balance between long-term disease control and functional preservation [23]. Early rehabilitation—including facial physiotherapy, vestibular training, and swallowing therapy—could optimize recovery [48]. Additional evidence supports the role of adjuvant stereotactic radiosurgery [73] and functional rehabilitation protocols for cranial nerve recovery [74]. Multidisciplinary care, integrating neurosurgery, otorhinolaryngology, radiation oncology, and neurorehabilitation, has been shown to significantly enhance both functional and quality-of-life outcomes [76].

#### 4.2.7. Future Perspectives

Emerging technologies are reshaping cranial nerve-centered strategies. Diffusion tractography enables preoperative mapping of nerve trajectories [9]. Augmented reality and high-definition endoscopy enhance intraoperative orientation [77]. Quantitative intraoperative monitoring may provide predictive intraoperative feedback, enabling more individualized, function-preserving surgery. Looking ahead, the integration of multimodal imaging, artificial intelligence-based tractography, and predictive analytics is expected to refine risk assessment and guide intraoperative navigation. Personalized surgical planning combining radiomic and molecular data may soon allow better balancing of oncological control and nerve preservation [73,78].

### 4.3. Paragangliomas

The review on PGL identified 388 cases with intradural extension over a total of 1506 PGL cases. Management was generally considered to be multidisciplinary (13/14 studies, 93%); all studies reported surgical management of the intradural component, with preoperative embolization in 12/14 (86%). Radiotherapy is an option mentioned in 10/14 studies (71%).

#### 4.3.1. Diagnostic Setting

Choice of treatment of intradural PGL involves balancing the natural morbidity of the disease and its treatment. Assessment of the genetics of PGL to foresee its aggressivity and potential of malignancy is paramount and mandatory in the diagnostic setting. Germline mutations (particularly SDHx genes such as SDHB, SDHD, etc.) are well recognized to influence PGL biology, including multifocality, metastatic potential, and familial risk. Genetic results provide (i) risk stratification, (ii) design of surveillance strategies for patients and relatives, and (iii) the goal and extent of surgery (for example, the need for complete tumor removal in SHDB mutations). Given these implications, contemporary practice guidelines advocate offering genetic testing to all patients with PGL [79,80].

Somatostatin receptor PET imaging (e.g., 68Ga-DOTATOC/DOTATATE PET) has high sensitivity for detecting PGLs and is particularly useful for whole-body staging, identifying multifocal disease, and distinguishing PGL from other skull base lesions [80]. PET imaging can also help select patients for peptide receptor radionuclide therapy (PRRT). The near absence of PET reporting in our review points out the temporal gap between the publication dates and the recent current routine adoption of modern PET techniques.

#### 4.3.2. Principles of Treatment

The management of PGL with intradural extension represents a challenge because intradural growth involves critical contact with cranial nerves, vascular structures, and brainstem. Contrary to vestibular schwannoma, PGL growth in the CPA has a high bleeding risk, which adds up to the intimate relationship with lower cranial nerves, whose preservation is rarely feasible. Most of the time, intradural growth is the effect of a big extradural mass infiltrating the medial wall of the jugular foramen with the cranial nerves and posterior cranial fossa dura, whose vessels feed the tumor. Clinicians are forced to balance tumor control and functional preservation.

Staged surgery—initially addressing the extracranial component together with the intracranial–extradural part—is often the preferred approach to achieve safe resection in both compartments [54,81]. Intradural extension into the cerebellopontine angle (CPA) is classified as Di1–2 according to Fisch [81], whereas Di3 was traditionally considered a condition of inoperability. However, even large intradural paragangliomas have been successfully managed with staged surgery and a combined resection strategy, in which pre-emptive coagulation of the dura of the posterior cranial fossa contributed to shrinking the highly vascular intradural mass [54]. The Di3 category has therefore been redefined to include large intradural tumors for which surgery remains feasible, thereby reducing the criteria for inoperability. Centralization of these cases in referral centers is also essential [82]. Angiography still plays a role in the preoperative setting to assess vascularity and perform angioembolization. The intradural PGL is scarcely treated with preoperative embolization, but the first stage extradural approach provides devascularization and better handling at the second stage intradural tumor resection [54].

In cases where surgical resection is not feasible despite adherence to the aforementioned principles, peptide receptor radionuclide therapy with (^177^Lu-DOTA^0^-Tyr^3^)octreotate (^177^Lu-DOTATATE) has been identified as a promising therapeutic approach [83].

#### 4.3.3. Cranial Nerve Morbidity in CPA PGL

Morbidity in PGL relates both to the natural history of the disease and surgical strategies. Cranial nerve disfunction (IX-X-XI- and XII) occurs slowly over the years in relation to the location in the jugular foramen and is better tolerated than sudden surgical morbidity for tumor resection. Cranial nerve status must be carefully assessed preoperatively because it both predicts surgical complexity and drives perioperative planning. In our dataset, all studies reported preoperative cranial nerve status and 13/14 (93%) also reported postoperative status, underscoring the centrality of nerve function monitoring in outcome reporting.

Surgical resection is the primary modality for controlling the intradural component of PGL and was reported in all included studies. The goals are maximal safe resection with no further loss except cranial nerve losses, whose preservation is hardly achievable, no different to the more frequent extradural JF paraganglioma.

The C1 extradural cases may be resected with preservation of the lower cranial nerves, facial nerve, and hearing with the more conservative petro-occipital-transigmoid approach [54,60], and a limited portion of Di can be resected without functional impact. Higher stage tumors, (C2, C3 with consistent intradural tumor growth) especially if with SDHB genetics, cannot be resected without sacrifice of the nerves. The facial nerve is generally preserved, but if the type A infratemporal approach [71] is performed, a slight impairment of the nerve after recovery can remain due to the nerve transposition. Similarly, conductive hearing loss is the unavoidable consequence of middle ear removal, which occurs at any case.

Postoperatively, early and targeted rehabilitation (facial reanimation strategies, vestibular rehabilitation, audiologic interventions including cochlear implants when applicable, comprehensive swallowing therapy, voice therapy, laryngeal and vocal cord treatment) can mitigate disability and improve functional recovery. Multidisciplinary rehabilitation teams (ENT, speech and language therapy, physiotherapy, neurorehabilitation specialists) should be engaged early. Age at surgery is paramount to plan and perform rehabilitation. In our review, the possible use of radiotherapy was reported in approximately 71% of studies. Radiotherapy, including stereotactic radiosurgery (SRS) and fractionated stereotactic radiotherapy, is an established option for residual, recurrent, or unresectable disease and for older patients and/or patients unfit for surgery. Nevertheless, the concept of unresectable diseases [54] is to be defined, and little experience is reported for exclusive radiotherapy treatment in intradural PGL, especially in wide extensions (Di2, Di3), where the increased risks of bleeding after radiation are to be carefully weighted.

## 5. Conclusions

The management of skull base tumors such as vestibular schwannomas, meningiomas, and paragangliomas requires balancing oncological control and functional preservation. In VS, SRS achieves high rates of tumor control and favorable facial nerve outcomes but carries specific risks, including trigeminal neuropathy and delayed hydrocephalus. Microsurgical resection remains a valid alternative and is particularly indicated for large or giant lesions producing mass effect.

In large and petroclival meningiomas, surgical objectives have shifted from radical excision to maximal safe resection, with an emphasis on cranial nerve preservation. Advances in imaging, intraoperative neurophysiological monitoring, and endoscopic assistance have significantly enhanced surgical safety. Staged procedures or combined strategies incorporating adjuvant radiosurgery are increasingly applied to optimize both tumor control and functional outcomes.

Paragangliomas with intradural extension pose distinct challenges due to their vascularity and cranial nerve involvement. Surgery is often staged and combined with preoperative embolization; it is addressed to the intradural tumor after previous extradural removal. Functional loss of the lower cranial nerves is common, both at diagnosis and after surgery, and is particularly likely when jugular foramen tumors extend into the CPA. Genetic testing and modern imaging modalities play a central role in risk stratification and management planning.

Across all three histologies, the current evidence highlights the importance of multidisciplinary teams, integrating neurosurgery, otorhinolaryngology, skull base surgery, neurotology, radiation oncology, neuroradiology, and rehabilitation. Rehabilitation—addressing swallowing, phonation, balance, and facial function—has become essential to achieving the dual goals of disease control and quality of life.

In large tumors, when the brainstem involvement reflects extensive disease due to tumor size and location, the contemporary paradigm emphasizes tumor control while prioritizing preservation and rehabilitation of function.

## Data Availability

No new data were created.
